# Immunosuppressive and monoclonal antibody treatment for myasthenia gravis: A network meta‐analysis

**DOI:** 10.1111/cns.13110

**Published:** 2019-02-26

**Authors:** Liang Wang, Xiao Huan, Jian‐Ying Xi, Hui Wu, Lei Zhou, Jia‐Hong Lu, Tian‐Song Zhang, Chong‐Bo Zhao

**Affiliations:** ^1^ Department of Neurology, Huashan Hospital Fudan University Shanghai China; ^2^ Department of Neurology, Jing’an District Centre Hospital of Shanghai Fudan University Shanghai China; ^3^ Department of Chinese Traditional Medicine, Jing’an District Centre Hospital of Shanghai Fudan University Shanghai China

**Keywords:** immunosuppressive agents, immunotherapy, monoclonal antibody, myasthenia gravis

## Abstract

**Background:**

We intended to compare and rank all the immunotherapies including immunosuppressant agents or monoclonal antibodies for myasthenia gravis (MG).

**Methods:**

A network meta‐analysis was performed to synthesize the direct evidence and indirect evidence. Quantitative MG score (QMGS) was defined as the primary outcome. The secondary outcomes included the glucocorticoid reduction and hazard ratios (HR) from the counts of adverse events (AEs).

**Results:**

We identified 14 studies including 808 MG patients. For the primary outcome, cyclosporine A (CsA) was hierarchically the best with statistical significances of −1.18 (−1.81, −0.59) vs placebo (PLA), −0.98 (−1.72, −0.23) vs mycophenolate mofetil, and −0.77 (−1.57, −0.032) vs tacrolimus (TAC). When the intervention periods were controlled, both eculizumab (ECZ) of −1.50 (−2.81, −0.18) and CsA of −1.23 (−1.81, −0.64) vs PLA reached a statistical significance. Belimumab and ECZ ranked the most tolerable therapies while CsA of 2.41 (0.58, 10.01) ranked the last vs PLA.

**Conclusion:**

These findings demonstrated that ECZ represented the most effective and tolerable therapeutic alternative to be recommended for refractory MG. TAC may be a beneficial therapy to treat MG extensively while the efficacy of CsA and cyclophosphamide may be limited by their multiple or severe AEs.

## 1 INTRODUCTION

Myasthenia gravis (MG) is the most common disorder of neuromuscular transmission resulting from antibodies to acetylcholine receptor (AChR), muscle‐specific kinase (MuSK), lipoprotein‐related protein 4, or other components in the postsynaptic membrane at the neuromuscular junction (NMJ).^1^, ^2^ This usually leads to characteristically fluctuating muscle weakness. The incidence of MG ranges from 1.7 to 21.3 million person‐years, and prevalence ranges from 15 to 179 per million persons.^3^

Standard therapy usually includes symptomatic therapy with cholinesterase inhibitors, immunosuppressive agents with glucocorticoids (GC), azathioprine (AZA), ciclosporin A (CsA), cyclophosphamide (CTX), methotrexate (MTX), tacrolimus (TAC), or mycophenolate mofetil (MMF).^4^ Despite lacking advanced evidence of evidence‐based interventions, GC and AZA remain the first‐line therapies. Thymectomy for MG may be an elective procedure while plasmapheresis (PP), immunoadsorption, and intravenous immunoglobulin (IVIg) produce rapid improvement in weakness. Recently, monoclonal antibodies are emerging therapeutic alternatives for MG. Monoclonal antibodies to CD20 (MabThera^TM^), C5b9 (Soliris^TM^) are beneficial drug to treat severe generalized MG.^5^, ^6^ So far, traditional meta‐analysis has evaluated the efficacy and safety of the above single or double intervention.^7^, ^8^, ^9^ However, none of them had included adequate assessment of comparative effectiveness of immunotherapies. Therefore, we performed a network meta‐analysis (NMA) of all relevant immunotherapies to comprehensively compare and rank strategies for MG treatment.

## 2 METHODS

### 2.1 Search strategy and selection criteria

**Figure 1:**
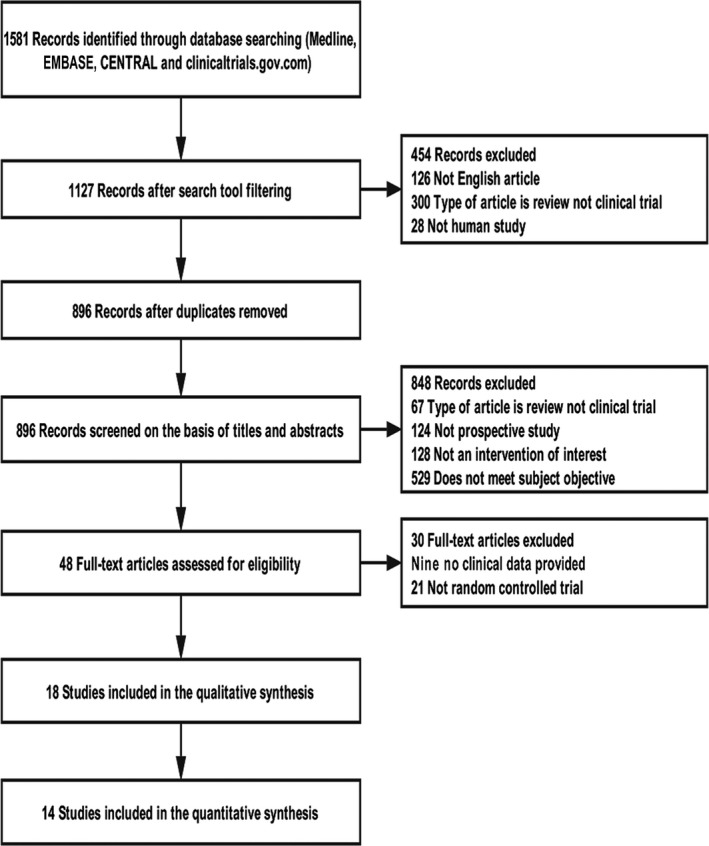
PRISMA chart of selection procedure and reasons for withdrawal from the network meta‐analysis. CENTRAL, Cochrane Central Register of Controlled Trials

Two reviewers (Liang Wang and Xiao Huan) independently searched Medline, Cochrane Central Register of Controlled Trials (CENTRAL), EMBASE, and clinicaltrials.gov databases. A search algorithm including a combination of relevant terms was employed for each database (Appendices 1, 2, 3, 4). The records were limited to randomized controlled trials (RCTs) including crossover trials published up to August 31, 2018, in English. We included all the relevant immunosuppressive agents and monoclonal antibodies. The treatment strategies of high‐dose methylprednisolone (HDMP), IVIg, PP, thymectomy, tirasemtiv, and terbutaline^2^ were excluded for their short‐term interventions. Two investigators (Liang Wang and Xiao Huan) read the studies integrally to evaluate the appropriate inclusion in the NMA. Any discrepancies were resolved by arbitration of a third investigator (Jian‐Ying Xi) to reach a consensus. The Preferred Reporting Items for Systematic Reviews and Meta‐Analyses (PRISMA) extension statement for NMA was followed, and its chart of search strategy was shown in Figure 1.

### 2.2 Data extraction and outcome measures

Information regarding study design, participant information, intervention or comparison method, and outcome measures was extracted when available. The extracted participant information included sample size, follow‐up months, age at onset, disease duration, number of thymectomy, number of thymoma, and number of anti‐AChR antibody serostatus.

Data were extracted by two reviewers (Liang Wang and Xiao Huan) with a standardized data extraction form and were inspected by another reviewer (Jian‐Ying Xi). We examined the published data provided in the original studies and contacted the corresponding authors for additional unpublished data. Any contradictory data were discussed and reached a consensus finally. For studies lacking change in standardization (SD), a method of single imputation with correlation coefficient was utilized.^10^

In this NMA, MG Foundation of America (MGFA) quantitative MG score (QMGS)^11^ was defined as the primary outcome. The secondary outcome included the steroid‐sparing effect measured by GC reduction. Another key secondary endpoint was the safety measured by drug‐related adverse events (AEs) in the follow‐up months. The MG activities of daily living (MG‐ADL),^12^ serum anti‐AChR antibody titer, and improvement rate were not included for the limited number of eligible studies.

### 2.3 Quality assessment‐risk of bias

The included studies were graded using the Oxford hierarchy of evidence 2011.^13^ The Cochrane risk of bias tool was employed to assess their risk of bias.^14^ Two reviewers (Liang Wang and Xiao Huan) evaluated the quality of studies and risk of bias independently. The criteria for evaluating the methodological quality included random sequence generation, allocation concealment, blinding of participants, blinding of observers, incomplete outcome data, selective reporting, and other bias. Comparison‐adjusted funnel plot was utilized to test the small‐study effect including publication bias.

### 2.4 Statistical analysis

Network meta‐analysis was performed with Bayesian Markov chain Monte Carlo model.^15^ We estimated relative treatment efficacy of the competing interventions by employing standardized mean differences (SMD) for continuous variables with 95% confidence intervals (CI). Besides, traditional pairwise meta‐analysis of random effects was conducted using “metan” command in each intervention, respectively. Random effects Poisson model was used to evaluate hazard ratios (HR) for count variables with 95% CI. We applied burn‐in phase of 40 000 iterations after 20 000 annealing algorithm to evaluate convergence. And surface under the cumulative ranking curve (SUCRA) was employed to acquire the efficacy hierarchy of competing interventions. Finally, we performed network meta‐regression with controlled intervention periods to figure out the genuine efficacy.

To check for the existence of inconsistency, the method of node‐splitting model by Dias^16^ was applied. The assumption of consistency was further verified by the design‐by‐treatment interaction model of Higgins.^17^ WinBUGS 1.4.3 (MRC Biostatistics Unit, Cambridge, UK), Stata 13.0 (StataCorp, College Station, TX, USA), and Revman 5.3 (Cochrane Collaboration, Oxford, UK) were utilized to perform this NMA.

## 3 RESULTS

### 3.1 Study characteristics

There were 1581 studies identified through database searching and other sources. Studies of two or more treatment arms with sufficient data were included in the quantitative synthesis. Considering the placebo effects, data from three trials without controlled placebo^18^, ^19^, ^20^ were excluded in the quantitative analysis. The data from the two studies by Sanders^21^, ^22^ were not pooled for different participant cohorts, neither were the data from the four studies by Howard^23^, ^24^ or Tindall^25^, ^26^ for different time points. Finally, 14 studies^21^, ^22^, ^23^, ^24^, ^25^, ^26^, ^27^, ^28^, ^29^, ^30^, ^31^, ^32^, ^33^, ^34^ were included in the NMA of our study.

In total, 808 MG patients from the combined datasets were included. The clinical and demographic characteristics were listed in Table 1. Different regimens and intervention periods were also summarized across the trials. The median sample size was 39 patients (range: 14‐176). Thymectomy was performed in 245 of 769 (31.9%) reported participants while thymoma was found in 48 of 390 (11.8%) reported participants. The anti‐AChR antibody serostatus was displayed in 725 patients, with 684 (94.3%) seropositive samples. CTX, MTX, eculizumab (ECZ), and belimumab (BLM) with corresponding placebo were administered intravenously while the other agents were taken orally. The median follow‐up time was 7.5 months (range: 3‐36 months).

**Table 1:** Randomized controlled trials and patient characteristics

Reference	Year	Interventions	Sample size (I/C)	Patients’ muscle involvement	Intervention periods (mo)	Age (I/C, y)	Time since onset (I/C, y)	Thymectomy (I/C)	Thymoma (I/C)	AChR+ (I/C)	Regimen	Outcome measures
TAC												
Zhou^28^	2017	TAC vs PLA	45/38	GMG	6	41.0/44.0	2.3/5.3	14	13	NA	3 mg/d po	1,2,3
Yoshikawa^29^	2011	TAC vs PLA	40/40	OMG: I/C = 14/11	7	45.9/44.4	7.4/7.9	28/30	12/18	28/29	3 mg/d po	1,2,3
				GMG: I/C = 26/29								
MMF												
Sanders^21^	2008a	MMF vs PLA	41/39	GMG	3	57.1/55.3	2.0/2.0	0/0	0/0	41/38	2.5 g/d po	1,2,3
Sanders^22^	2008b	MMF vs PLA	88/88	GMG	9	49.0/49.7	2.8/3.3	23/25	NA	88/88	2 g/d po	1,3
Meriggioli^27^	2003	MMF vs PLA	7/7	GMG	5	57.7/51.3	9.0/9.9	3/5	0/0	5/6	2 g/d po	1,3
CsA												
Tindall^25^	1993	CsA vs PLA	20/19	GMG	6	56.1/43.7	NA	NA	NA	20/19	5 mg/kg/d po	1,2
Tindall^26^	1987	CsA vs PLA	10/10	GMG	12	64.0/66.2	NA	0/0	NA	10/10	6 mg/kg/d po	1,3
CTX												
De Feo^33^	2002	CTX vs PLA	12/11	GMG	12	44.2/39.9	NA	8/8	NA	12/11	500 mg/m^2^/mo iv	2,3
MTX												
Pasnoor^31^	2016	MTX vs PLA	25/25	GMG	12	66.5/68.6	NA	0/0	0/0	25/25	20 mg/wk iv	1,2,3
Heckmann^32^	2011	MTX vs AZA	16/15	GMG	24	47.9/42.7	7.5/10.3	2/1	3/2	10/9	17.5 mg/wk iv vs	1,2,3
											2.5 mg/kg/d po	
AZA												
Palace^30^	1998	AZA vs PLA	15/19	GMG	36	58.5/55.7	2.3/2.3	6/5	0/2	15/19	2.5 mg/kg/d po	2,3
ECZ												
Howard^24^	2017	ECZ vs PLA	62/63	GMG	6.5	38.0/38.1	9.9/9.2	37/31	NA	62/63	900 mg/wk‐1200 mg/2 wk iv	1,3
Howard^a^, ^23^	2013	ECZ vs PLA	14	GMG	8	48	7	6	0	14	600 mg/wk‐900 mg/2 wk iv	1,3
BLM												
Hewett^34^	2018	BLM vs PLA	18/21	GMG	6	52.7/59.0	8.7/9.0	6/7	0/0	18/19	10 mg/kg/mo iv	1,3

Outcome measures: 1. The reduction of quantitative myasthenia gravis score; 2. The reduction of glucocorticoids; 3. Adverse events. Values are n or mean.

AChR, acetylcholine receptor; AZA, azathioprine; BLM, belimumab; C, control; CsA, cyclosporine A; CTX, cyclophosphamide; ECZ, eculizumab; GMG, generalized myasthenia gravis; I, intervention; iv, intravenous; MMF, mycophenolate mofetil; MTX, methotrexate; NA, not available; OMG, ocular myasthenia gravis; PLA, placebo; Po, peros; TAC, tacrolimus.

a Corssover randomized controlled trial.

### 3.2 Efficacy comparison on the QMGS

**Figure 2:**
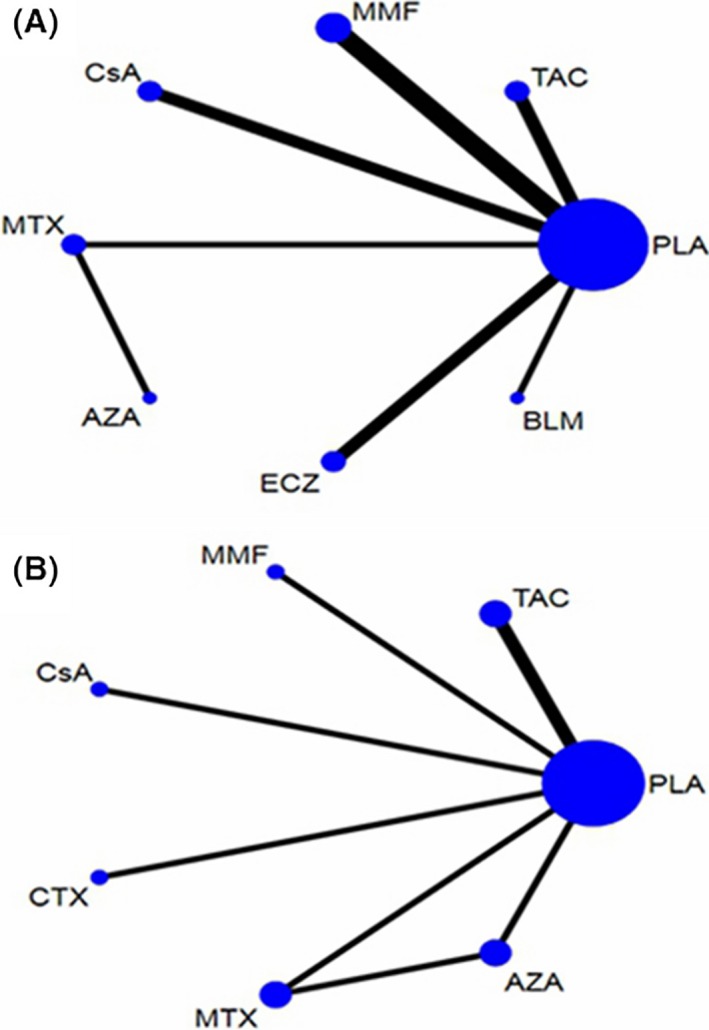
A, Network of treatment comparisons for the primary outcome of quantitative myasthenia gravis score. B, Network of treatment comparisons for the secondary outcome of glucocorticoid reduction. The size of nodes is in proportion to the number of trials that assessed the same intervention and the thickness of lines corresponds to the number of trials which have a direct comparison. AZA, azathioprine; BLM, belimumab; CsA, cyclosporine A; CTX, cyclophosphamide; ECZ, eculizumab; MMF, mycophenolate mofetil; MTX, methotrexate; PLA, placebo; PLA, placebo; TAC, tacrolimus

**Figure 3:**
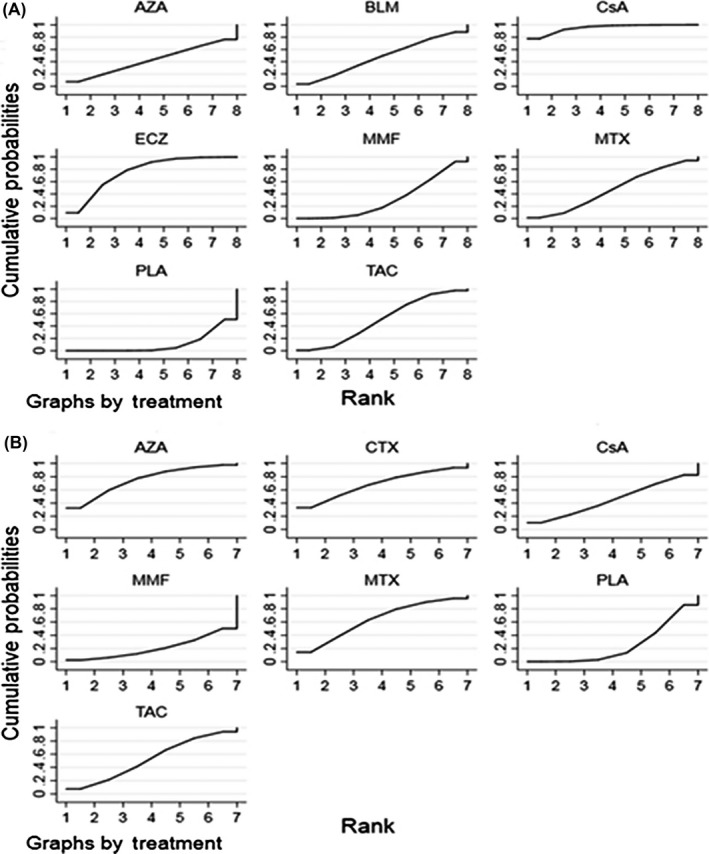
A, Surface under the cumulative ranking curve of available competing interventions for quantitative myasthenia gravis score. B, Surface under the cumulative ranking curve of available competing interventions for glucocorticoid reduction. PLA, placebo; TAC, tacrolimus; MMF, mycophenolate mofetil; CsA, cyclosporine A; CTX, cyclophophamide; MTX, methotrexate; AZA, azathioprine; ECZ, eculizumab; BLM, belimumab

**Figure 4:**
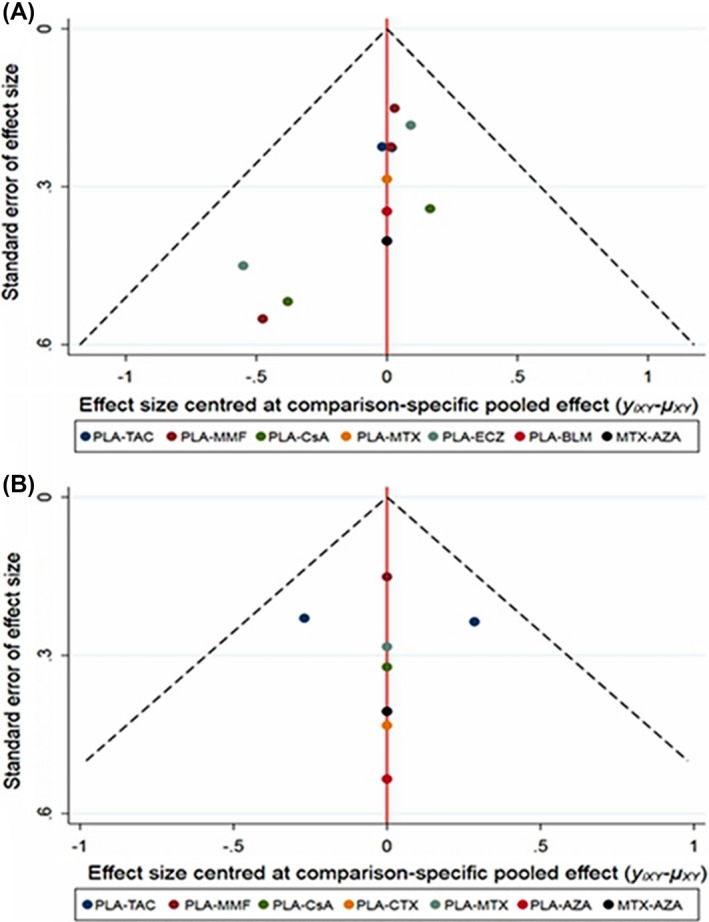
A, Comparison‐adjusted funnel plot for the primary outcome of quantitative myasthenia gravis score. B, Comparison‐adjusted funnel plot for the secondary outcome of glucocorticoid reduction. The red line is representative of the null hypothesis that the study‐specific effect sizes are not different from the respective comparison‐specific summary estimates. The two black dashed lines are representative of a 95% CI for the difference between comparison‐specific pool effect estimates and study‐specific effect sizes. *y_ixy_* is the noted effect size for *x* vs *y* in study *i*. *μ_xy_* is the comparison‐specific summary estimate that compares *x* with *y*. AZA, azathioprine; BLM, belimumab; CsA, cyclosporine A; CTX, cyclophosphamide; ECZ, eculizumab; MMF, mycophenolate mofetil; MTX, methotrexate; PLA, placebo; TAC, tacrolimus; MMF, mycophenolate mofetil; CsA, cyclosporine A; CTX, cyclophophamide; MTX, methotrexate; AZA, azathioprine; ECZ, eculizumab; BLM, belimumab

There were 12 studies involving eight interventions including immunosuppressive agents and monoclonal antibodies evaluating the reduction of QMGS. The network plot was shown in Figure 2A, and estimated SMDs of the relative efficacy are shown in Table 2 with median values and 95% CI. With traditional pairwise mean‐analysis, statistical significances were calculated in CsA of −1.19 (−1.75, −0.63) vs PLA, ECZ of −0.80 (−1.37, −0.23) vs PLA, and TAC of −0.41 (−0.72 to −0.096) vs PLA. According to SUCRA, CsA was hierarchically the best, with statistical significances of −1.18 (−1.81, −0.59) vs PLA, −0.98 (−1.72, −0.23) vs MMF, and −0.77 (−1.57, −0.032) vs TAC. ECZ was ranked second with statistical significances of −0.75 (−1.33, −0.30) vs PLA while TAC was ranked third of −0.41 (−0.88, 0.065; Figure 3A). BLM, MTX, AZA, and MMF were not demonstrated to be efficacious. Additionally, improved muscle strength with statistical significance (*P* < 0.025) was reported using CTX although QMGS was not conducted. For the loop was not formed in the primary outcome, there was no source of inconsistency. Comparison‐adjusted funnel plot was shown in Figure 4A and revealed possible small‐study effects for the QMGS. Network meta‐regression was further conducted. When the follow‐up months were controlled, ECZ of −1.50 (−2.81, −0.18) vs PLA and CsA of −1.23 (−1.81, −0.64) vs PLA reached a statistical significance in the QMGS.

**Table 2:** Estimated differences in the efficacy of interventions on quantitative myasthenia gravis score

	Standardized mean difference using traditional pairwise meta‐analysis							
Standard ized mean difference with network meta‐analysis	Cyclosporine A	—	—	—	—	—	—	**−1.19 (−1.75, −0.63)**
	**−**0.42 (**−**1.19, 0.40)	Eculizumab	**—**	**—**	**—**	**—**	**—**	**−0.80 (−1.37, −0.23)**
	**−0.77 (−1.57, −0.032)**	**−**0.34 (**−**1.11, 0.29)	Tacrolimus	**—**	**—**	**—**	**—**	**−0.41 (−0.72, −0.096)**
	**−**0.78 (**−**1.85, 0.22)	**−**0.37 (**−**1.36, 0.59)	**−**0.014 (**−**0.95, 0.95)	Belimumab	**—**	**—**	**—**	**−**0.40 (**−**1.08, 0.28)
	**−**0.79 (**−**1.78, 0.14)	**−**0.37 (**−**1.31, 0.47)	**−**0.024 (**−**0.90, 0.85)	**−**0.012 (**−**1.14, 1.09)	Methotrexate	**—**	**—**	**−**0.39 (**−**0.94, 0.18)
	**−**0.86 (**−**2.18, 0.49)	**−**0.45 (**−**1.73, 0.86)	**−**0.090 (**−**1.34, 1.24)	**−**0.084 (**−**1.52, 1.45)	**−**0.058 (**−**0.98,0.92)	Azathioprine	0.041 (**−**0.75, 0.83)	**—**
	**−0.98 (−1.72, −0.23)**	**−**0.56 (**−**1.24, 0.062)	**−**0.22 (**−**0.80, 0.45)	**−**0.19 (**−**1.10, 0.74)	**−**0.19 (**−**0.99, 0.67)	**−**0.12 (**−**1.41, 1.13)	Mycophenolate mofetil	**−**0.17 (**−**0.41, 0.066)
	**−1.18 (−1.81, −0.59)**	**−0.75 (−1.33, −0.30)**	**−**0.41 (**−**0.88, 0.065)	**−**0.39 (**−**1.23, 0.43)	**−**0.38 (**−**1.11, 0.36)	**−**0.32 (**−**1.56, 0.83)	**−**0.19 (**−**0.64, 0.17)	Placebo

Median values of standardized mean differences with 95% confidence intervals (column vs row) of the efficacy of interventions are exhibited on the lower left part of the table while standardized mean differences with 95% confidence intervals using metan command are exhibited on the upper right of the table. Values lower than zero favor the column‐defining intervention. Interventions are ordered in accordance with efficacy ranking. Numbers in bold with darker shades show statistically significant results.

### 3.3 Efficacy comparison on the reduction of GC

Eight studies evaluating the reduction of GC with seven immunosuppressive agents were included in this NMA. Figure 2B revealed the network plot while Table 3 listed the estimated SMDs of the relative efficacy with median value and 95% CI, agent by agent. Compared with PLA, only AZA therapy lasting 36 months demonstrated to be statistically efficacious (*P* = 0.009) while a correlation trend was shown in CTX (*P* = 0.086). When using SUCRA (Figure 3B), AZA was ranked the best treatment while CTX was hierarchically the second. However, inconsistency existed in AZA vs PLA with the design‐by‐treatment interaction model (*P* = 0.032) while not significant in the node‐splitting model (*P* = 0.104). Besides, Figure 4B exhibited the absence of small‐study effects for GC reduction. We further employed network meta‐regression to control the intervention periods. However, compared with PLA, the statistical differences were not significant in any immunosuppressive agents.

**Table 3:** Estimated differences in the efficacy of interventions on glucocorticoid reduction

	Standardized mean difference using traditional pairwise meta‐analysis						
Stand ardized mean difference with network meta‐analysis	Azathioprine	—	0.35 (−0.44, 1.15)	—	—	−**1.39 (**−**2.44, **−**0.35)**	—
	−0.072 (−1.97, 1.73)	Cyclophosphamide	—	—	—	−0.74 (−1.59, 0.11)	—
	−0.20 (−1.36, 0.99)	−0.13 (−1.89, 1.72)	Methotrexate	—	—	−0.19 (−0.75, 0.36)	—
	−0.41 (−1.92, 1.03)	−0.33 (−2.05, 1.37)	−0.20 (−1.70, 1.16)	Tacrolimus	—	−0.38 (−0.92, 0.17)	—
	−0.51 (−2.32, 1.23)	−0.44 (−2.43, 1.55)	−0.31 (−2.10, 1.38)	−0.10 (−1.73, 1.51)	Cyclosporine A	−0.28 (−0.91, 0.35)	—
	−0.79 (−1.98, 0.34)	−0.71 (−2.14, 0.72)	−0.58 (−1.75, 0.48)	−0.38 (−1.29, 0.55)	−0.27 (−1.60, 1.09)	Placebo	−0.16 (−0.46, 0.13)
	−0.94 (−2.67, 0.73)	−0.87 (−2.77, 1.04)	−0.75 (−2.47, 0.89)	−0.54 (−2.09, 1.01)	−0.44 (−2.25, 1.41)	−0.17 (−1.43, 1.09)	Mycophenolate mofetil

Median values of standardized mean differences with 95% confidence intervals (column vs row) of the efficacy of interventions are exhibited on the lower left part of the table while standardized mean differences with 95% confidence intervals using metan command are exhibited on the upper right of the table. Values lower than zero favor the column‐defining intervention. Interventions are ordered in accordance with efficacy ranking. Numbers in bold with darker shades show statistically significant results.

### 3.4 Safety comparison of AEs

Adverse events were counted during the intervention combined with the number of participants, respectively. Relative median values with 95% CI were exhibited using HR with random effects Poisson model to control the time and number (Table 4). BLM and ECZ ranked the most tolerable therapies causing the least counts of AEs while CsA of 2.41 (0.58, 10.01) ranked the last vs PLA, implicating the most counts of AEs. Additionally, the counts of AEs in the other immunotherapies did not differ significantly. Although the exact number of AEs could not be acquired from the study about CTX, the incidence between CTX and PLA groups did not show statistical difference.

**Table 4:** Estimated hazard ratios of interventions on adverse events

Hazard ratio	Belimumab	—	—	—	—	—	—	—
	1.09 (0.22,6.70)	Eculizumab	—	—	—	—	—	—
	1.16 (0.28, 4.80)	1.03 (0.36, 2.64)	Placebo	—	—	—	—	—
	1.20 (0.11, 11.5)	1.05 (0.11, 8.30)	1.03 (0.15, 6.77)	Azathioprine	—	—	—	—
	1.25 (0.19, 8.46)	1.14 (0.21, 5.53)	1.10 (0.29, 4.13)	1.09 (0.28, 4.29)	Methotrexate	—	—	—
	1.31 (0.23, 7.31)	1.18 (0.27, 4,31)	1.13 (0.41, 2.97)	1.09 (0.13, 9.24)	1.04 (0.20, 5.41)	Tacrolimus	—	—
	1.46 (0.42, 12.61)	1.32 (0.49, 7.77)	1.25 (0.78, 5.29)	1.21 (0.25, 16.6)	1.15 (0.38, 9.60)	1.10 (0.46, 7.27)	Mycophenolate mofetil	—
	2.81 (0.38, 20.73)	2.48 (0.42, 13.54)	2.41 (0.58, 10.01)	2.36 (0.23, 26.29)	2.20 (0.32, 15.27)	2.11 (0.38, 12.59)	1.89 (0.22, 6.78)	Cyclosporine A

Median values of hazard ratios with 95% confidence intervals (column vs row) of the safety of interventions are exhibited on the lower left part of the table. Values upper than one favor the column‐defining intervention. Interventions are ordered in accordance with safety ranking.

### 3.5 Risk of bias

**Figure 5:**
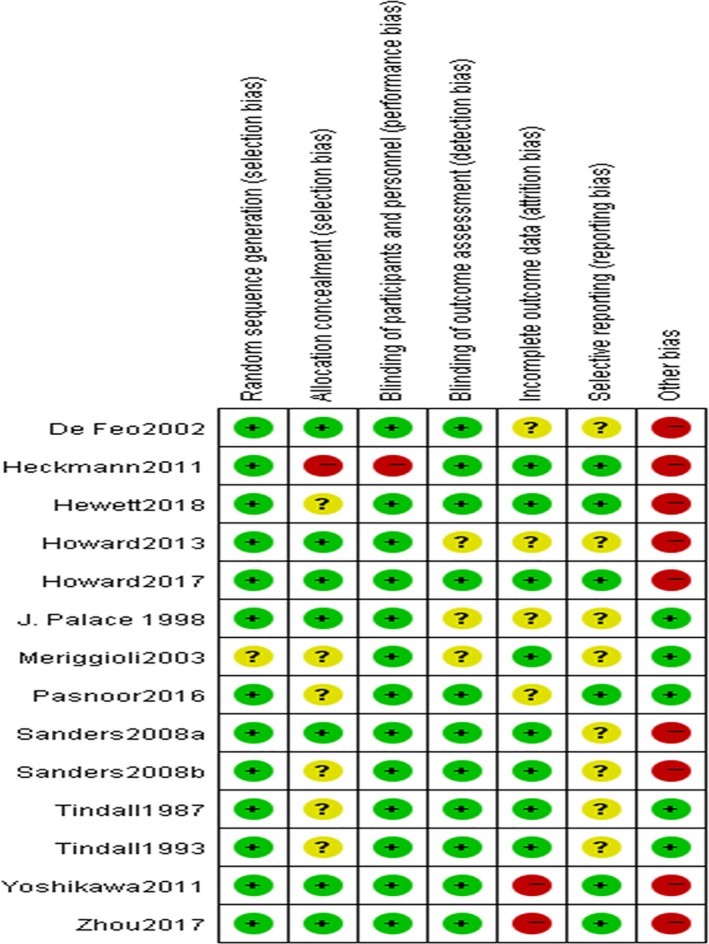
Risk of bias summary: reviewers’ judgments for each included trial about each risk of bias item

All of the included studies were graded as level 2 for their randomized design. However, it is difficult to assess the risk of bias for the lack of detailed reporting. The overall quality of included studies shown in Figure 5 was moderate to low. Studies were not identified in random sequence generation and selecting reporting with definite high risk of bias. One study had evidence of allocation concealment, and two studies exhibited high risk for incomplete outcome data. Two studies were not definitely blinding in participants while three were not clearly defined in detection blinding. Besides, we identified eight studies with definite high risk of other bias including carryover effect, recruitment bias, and publication bias.

## 4 DISCUSSION

This NMA including 808 patients across 14 RCTs represented the most comprehensive data analysis for current immunotherapies for MG. Principal findings were as follows: First, ECZ represented the most effective therapeutic alternative to improve QMGS with good tolerability, which could be recommended in the refractory MG patients. Second, TAC may be a beneficial therapy to extensively treat MG with relatively favorable results while the efficacy of CsA and CTX could be limited by their multiple or severe AEs. Third, the efficacy of AZA, MMF, MTX, and BLM may not be significant for MG treatment.

Our findings concluded that ECZ could be the most efficacious strategy for MG treatment with minor AEs. Complement activation at the NMJ may be the principal cause of AChR loss and blockade of neuromuscular transmission observed in MG.^5^ As a humanized monoclonal antibody, ECZ could specifically bind to human terminal complement protein C5 with high affinity to inhibit the formation of proteins C5a and C5b through enzymatic cleavage. The C5a‐induced chemotaxis of proinflammatory cells could be restrained as well as the C5b‐induced membrane attack complex.^35^ ECZ was recommended in complement‐mediated diseases of atypical hemolytic uremic syndrome, paroxysmal nocturnal hemoglobinuria, and refractory generalized MG which could be unresponsive to GC and at least two immunosuppressive agents.^24^, ^36^ Notably, ECZ was employed in the two RCTs (ie, class I, Level A) for refractory generalized MG which may undervalue its genuine efficacy. Compared with PLA in QMGS, a significant difference was calculated both in the NMA (−0.75, −1.33 to −0.30) and in the traditional pairwise meta‐analysis (−0.80, −1.37 to −0.23), with one of the lowest HR of AEs even superior to PLA (1.16, 0.28 to 4.80). Its efficacy was hierarchically the best vs PLA (−1.50, −2.81 to −0.18) when the intervention periods were controlled. Although the reduction of GC was not reported in the RCTs, a steroid‐sparing effect of ECZ was suggested during the extension study.^37^ ECZ also comes into effect quickly and has long‐standing clinical benefits when compared with other immunosuppressive agents requiring several months to act.^38^ Therefore, inhibition of complement might be a superactive therapy for MG.

CsA and TAC both belong to calcineurin inhibitors (CNIs) while TAC exhibits 10‐100 more potency as well as less incidence of AEs such as renal toxicity and hypertension.^39^ CNIs were initially used in the organ transplantations and exhibited clinical benefit in the autoimmune diseases including systematic lupus erythematous (SLE). CsA was recommended in MG patients intolerable of AZA and MMF while TAC was suggested if they cannot tolerate CsA further.^40^ CNIs could inhibit the calcineurin activity as well as the transcription of inflammatory cytokines like interleukin‐2, preventing T cell from activation and B cell from antibody production.^41^ Besides, CNIs were demonstrated to restrain humoral immunity through acting directly on naïve B cells and inhibit plasmablast differentiation.^42^ Compared with PLA in QMGS, statistical significance was observed in both CsA (−1.19, −1.75 to −0.63) and TAC (−0.41, −0.72 to −0.096) using pairwise meta‐analysis. As for NMA, however, cyclosporine exhibited statistical significance over TAC (−1.18, −1.81 to −0.59) and PLA (−0.77, −1.57 to −0.032). When controlling the follow‐up months in the network meta‐regression, statistical difference in CsA vs PLA was still significant. This paradox may be attributed to the regimen: TAC was administered 3 mg/d orally while CsA 5‐6 mg/kg/d orally. This could also explain the reason why AEs occurred more frequently in CsA (2.11, 0.38 to 12.59) than TAC. Some people in the CsA group was withdrawn from the study due to the nephrotoxicity while this event was relatively mild in the TAC group. In addition, both TAC and CsA exhibited steroid‐sparing effect but the difference was not significant in our study mainly due to the short‐term follow‐up.

As an alkylating agent, CTX is widely applied in severe autoimmune disorders including MG.^42^ The hematopoietic and immune system could be repopulated with endogenous stem cells after intermittent high‐dose CTX.^6^ Although QMGS was not performed, muscle strength was improved with statistical significance. Significant reduction of GC was acquired from the study but not in this NMA or traditional pairwise meta‐analysis. The incidence of AEs between CTX and PLA did not reveal statistical significance. However, compared with PLA, CTX usually induces more severe AEs including diarrhea, hemorrhagic cystitis, infection, and infertility with carcinogenic, teratogenic potential.^6^

The other four immunosuppressive agents, AZA, MMF, MTX, and BLM were not so efficacious as the above agents. AZA exhibits the steroid‐sparing effect against PLA (−1.39, −2.44 to −0.35) in the 36 months of intervention while the difference was one of the least significant when employing the network meta‐regression. As the recommended first‐line steroid‐sparing agent, it could be more effective and tolerable when AZA was combined with GC than GC alone.^30^ The inconsistency from design‐by‐treatment interaction model (*P* = 0.032) above may be originated from the different follow‐up months. A long‐term AZA intervention was needed to induce significance. Although its AEs between AZA and PLA did not reveal statistical significance in this NMA, the potential for myelosuppression and hepatotoxicity is not uncommon.^43^ MMF, the first‐line steroid‐sparing agent widely used in USA, is also a purine antimetabolite that inhibits T‐cell and B‐cell proliferation like AZA.^40^ However, statistical significance was not found in the primary or secondary outcome measure. Neither was intravenous MTX which blocks pyrimidine and purine synthesis. MTX was comparable with AZA in the primary (−0.058, −0.98 to 0.92) and secondary (0.20, −0.99 to 1.36) outcome measures including safety in HR (1.09, 0.28 to 4.29). BLM, a human monoclonal antibody against B‐cell activating factor (BAFF),^34^ is an effective therapy for SLE. It was demonstrated that the level of CD19 + BAFFR + B cells was elevated in MG patients, verifying the increased activation of B‐cell maturation.^44^ However, the outcome measures of GC and QMGS reduction were negative, also the risk of suffering AEs was the lowest.

Unfortunately, rituximab (RTX), a mouse/human monoclonal antibody against CD20 antigen, was not included to evaluate its therapeutic effect in this NMA for the lack of RCTs. Intravenous RTX was recommended in refractory MG.^40^ One clinical controlled trial demonstrated the probability of favorable outcomes increases with a significant steroid‐sparing effect in anti‐MuSK antibody‐positive MG patients.^45^ A meta‐analysis of RTX concluded the overall effective rate was 83.9%, and incidence of AEs was rather low.^7^

There were several limitations from the evidence and NMA process: First, IVIg, PP, or HDMP was not restricted when the MG crisis happened while some immunosuppressants were administered during the monoclonal antibody therapy, so the specific therapeutic response could not be evaluated properly. Second, some data from these outcome measures were not listed individually so we had to use the estimated value. AEs were counted but severe AEs were not distinguished, leading to the tolerability calculated unproperly. Third, the including criteria of MG patients in different trials were not well controlled as well as the treatment duration, which could also influence the efficacy. Short‐term intervention may limit the steroid‐sparing effect. Moreover, our findings came from the direct and indirect comparisons in NMA model based on relative treatment effects. The reliability of demonstration was limited by few studies and closed loops per comparison. There were many factors which could influence the sensitivity of clinical trials.^46^ Although from RCTs, the primary outcome was not demonstrated to be improved in some agents, but they were still employed and showed efficacy in clinic. More reasonable clinical trials from the real world should be designed to explore their genuine efficacy and tolerability.

## 5 CONCLUSIONS

This comprehensive NMA concluded ECZ represented the most effective therapeutic alternative to improve QMGS with good tolerability, which could be recommended in the refractory MG patients. TAC may be a beneficial therapy to extensively treat MG with relatively favorable results while the efficacy of CsA and CTX could be limited by their multiple or severe AEs. The efficacy of AZA, MMF, MTX, and BLM may not be significant for MG treatment.

## CONFLICT OF INTEREST

The authors declare that they have no conflict of interest.

## APPENDIX 1

### Ovid MEDLINE Search Strategy

1. myasthenia gravis.mp. or exp Myasthenia Gravis/
2. myasthen$.mp. [mp=title, original title, abstract, name of substance word, subject heading word]
3. 1 or 2
4. azathioprine.mp. or Azathioprine/
5. cyclosporin A.mp. or Cyclosporins/
6. Cyclosporine/or cyclosporine A.mp.
7. ciclosporin.mp.
8. cyclophosphamide.mp. or Cyclophosphamide/
9. methotrexate.mp. or Methotrexate/
10. mycophenolate mofetil.mp.
11. tacrolimus.mp. or Tacrolimus/
12. FK‐506.mp.
13. leflunomide.mp. or Leflunomide/
14. rituximab.mp. or Rituximab/
15. belimumab.mp. or Belimumab/
16. eculizumab.mp. or Eculizumab/
17. ofatumumab.mp. or Ofatumumab/
18. etanercept.mp. or Etanercept/
19. infliximab.mp. or Infliximab/
20. adalimumab.mp. or Adalimumab/
21. daclizumab.mp. or Daclizumab/
22. natalizumab.mp. or Natalizumab/
23. tocilizumab.mp. or Tocilizumab/
24. brodalumab.mp. or Brodalumab/
25. ixekizumab.mp. or Ixekizumab/
26. daratumumab.mp. or Daratumumab/
27. brodalumab.mp. or Brodalumab/
28. secukinumab.mp. or Secukinumab/
29. immunosuppressive drug.mp.
30. immunosuppressive agent.mp. or Immunosuppressive Agents/
31. immunosuppression.mp. or Immunosuppression/
32. or/4‐31
33. randomized controlled trial.pt.
34. controlled clinical trial.pt.
35. randomized controlled trials/
36. random allocation/
37. double‐blind method/
38. single‐blind method/
39. or/33‐38
40. animals/not humans/
41. 39 not 40
42. clinical trial.pt.
43. exp clinical trial/
44. (clin$ adj25 trial$).ti,ab.
45. ((singl$ or doubl$ or tripl$ or trebl$) adj25 (blind$ or mask$)).ti,ab.
46. placebos/
47. placebo$.ti,ab.
48. random$.ti,ab.
49. research design/
50. or/42‐49
51. 50 not 40
52. 51 not 41
53. comparative study/
54. exp evaluation studies/
55. follow up studies/
56. prospective studies/
57. (control$ or prospectiv$ or volunteer$).ti,ab.
58. or/53‐57
59. 58 not 40
60. 59 not (41 or 42)
61. 41 or 52 or 60
62. 3 and 32 and 61

## APPENDIX 2

### Ovid EMBASESearch Strategy

1. myasthenia gravis.mp. or exp Myasthenia Gravis/
2. myasthen$.mp. [mp=title, abstract, subject headings, heading word, drug trade name, original title, device manufacturer, drug manufacturer name]
3. 1 or 2
4. azathioprine.mp. or Azathioprine/
5. cyclosporin A.mp. or Cyclosporins/
6. Cyclosporine/or cyclosporine A.mp.
7. ciclosporin.mp.
8. cyclophosphamide.mp. or Cyclophosphamide/
9. methotrexate.mp. or Methotrexate/
10. mycophenolate mofetil.mp.
11. tacrolimus.mp. or Tacrolimus/
12. FK‐506.mp.
13. leflunomide or Leflunomide/
14. rituximab.mp. or Rituximab/
15. belimumab.mp. or Belimumab/
16. eculizumab.mp. or Eculizumab/
17. ofatumumab.mp. or Ofatumumab/
18. etanercept.mp. or Etanercept/
19. infliximab.mp. or Infliximab/
20. adalimumab.mp. or Adalimumab/
21. daclizumab.mp. or Daclizumab/
22. natalizumab.mp. or Natalizumab/
23. tocilizumab.mp. or Tocilizumab/
24. brodalumab.mp. or Brodalumab/
25. ixekizumab.mp. or Ixekizumab/
26. daratumumab.mp. or Daratumumab/
27. brodalumab.mp. or Brodalumab/
28. secukinumab.mp. or Secukinumab/
29. immunosuppressive drug.mp.
30. immunosuppressive agent.mp. or Immunosuppressive Agents/
31. immunosuppression.mp. or Immunosuppression/
32. or/4‐29
33. Randomized Controlled Trial/
34. Clinical Trial/
35. Multicenter Study/
36. Controlled Study/
37. Crossover Procedure/
38. Double Blind Procedure/
39. Single Blind Procedure/
40. exp RANDOMIZATION/
41. PLACEBO/
42. Meta Analysis/
43. phase 2 clinical trial/or phase 3 clinical trial/or phase 4 clinical trial/
44. ((singl$ or doubl$ or tripl$ or trebl$) adj25 (blind$ or mask$)).tw.
45. placebo$.tw.
46. random$.tw.
47. control$.tw.
48. (meta?analys$ or systematic review$).tw.
49. (cross?over or factorial or sham? or dummy).tw.
50. ABAB design$.tw.
51. or/33‐50
52. human/
53. nonhuman/
54. 52 or 53
55. 51 not 54
56. 51 and 52
57. 55 or 56
58. 3 and 32 and 57

## APPENDIX 3

### Cochrane Central Register of Controlled Trials (CENTRAL) Search Strategy

#1. myasthenia gravis (in Trials (Word variations have been searched))
#2. azathioprine (in Trials (Word variations have been searched))
#3. cyclosporine (in Trials (Word variations have been searched))
#4. cyclophosphamide (in Trials (Word variations have been searched))
#5. methotrexate (in Trials (Word variations have been searched))
#6. mycophenolate mofetil (in Trials (Word variations have been searched))
#7. tacrolimus (in Trials (Word variations have been searched))
#8. FK‐506 (in Trials (Word variations have been searched))
#9. leflunomide(in Trials (Word variations have been searched))
#10 rituximab(in Trials (Word variations have been searched))
#11. belimumab (in Trials (Word variations have been searched))
#12. eculizumab (in Trials (Word variations have been searched))
#13. ofatumumab (in Trials (Word variations have been searched))
#14. etanercept (in Trials (Word variations have been searched))
#15. infliximab (in Trials (Word variations have been searched))
#16. adalimumab (in Trials (Word variations have been searched))
#17. daclizumab (in Trials (Word variations have been searched))
#18. natalizumab (in Trials (Word variations have been searched))
#19. tocilizumab (in Trials (Word variations have been searched))
#20. brodalumab (in Trials (Word variations have been searched))
#21. ixekizumab (in Trials (Word variations have been searched))
#22. daratumumab (in Trials (Word variations have been searched))
#23. brodalumab (in Trials (Word variations have been searched))
#24. secukinumab (in Trials (Word variations have been searched))
#25. immunosuppressive drug (in Trials (Word variations have been searched))
#26. immunosuppressive agent (in Trials (Word variations have been searched))
#27. immunosuppression (in Trials (Word variations have been searched))
#28. #2 or #3 or #4 or #5 or #6 or #7 or #8 or #9 or #10 or #11 or #12 or #13 or #14 or #15 or #16 or #17 or #18 or #19 or #20 or #21 or #22 or #22 or #23 or #24 or #25 or #26 or #27
#29. randomized controlled trial.pt.
#30. controlled clinical trial.pt.
#31. randomized controlled trials/
#32. random allocation/
#33. double‐blind method/
#34. single‐blind method/
#35. #29 or #30 or #31 or #32 or #33 or #34
#36. #1 and #28 and #35

## APPENDIX 4

### Clinicaltrials.gov Search Strategy

1. Myasthenia Gravis
